# Evaluating the Reliability and Accuracy of an AI-Powered Search Engine in Providing Responses on Dietary Supplements: Quantitative and Qualitative Evaluation

**DOI:** 10.2196/78436

**Published:** 2025-10-29

**Authors:** Mingxin Liu, Tsuyoshi Okuhara, Ritsuko Shirabe, Yuriko Nishiie, Yinghan Xu, Hiroko Okada, Takahiro Kiuchi

**Affiliations:** 1 Department of Health Communication, Graduate School of Medicine, The University of Tokyo, Bunkyo, Hongo 7-3-1, Tokyo, 113-8655, Japan, 81 03-5800-6549; 2 Department of Health Communication, School of Public Health, Graduate School of Medicine, The University of Tokyo, Tokyo, Japan; 3 University Hospital Medical Information Network (UMIN) Center, University of Tokyo Hospital, Tokyo, Japan; 4 Graduate School of Human Sciences, Waseda University, Tokorozawa, Japan

**Keywords:** artificial intelligence search engine, AI search engine, Copilot, dietary supplements, health communication, health education, large language model, artificial intelligence, AI

## Abstract

**Background:**

The widespread adoption of artificial intelligence (AI)–powered search engines has transformed how people access health information. Microsoft Copilot, formerly Bing Chat, offers real-time web-sourced responses to user queries, raising concerns about the reliability of its health content. This is particularly critical in the domain of dietary supplements, where scientific consensus is limited and online misinformation is prevalent. Despite the popularity of supplements in Japan, little is known about the accuracy of AI-generated advice on their effectiveness for common diseases.

**Objective:**

We aimed to evaluate the reliability and accuracy of Microsoft Copilot, an AI search engine, in responding to health-related queries about dietary supplements. Our findings can help consumers use large language models more safely and effectively when seeking information on dietary supplements and support developers in improving large language models’ performance in this field.

**Methods:**

We simulated typical consumer behavior by posing 180 questions (6 per supplement × 30 supplements) to Copilot’s 3 response modes (creative, balanced, and precise) in Japanese. These questions addressed the effectiveness of supplements in treating 6 common conditions (cancer, diabetes, obesity, constipation, joint pain, and hypertension). We classified the AI search engine’s answers as “effective,” “uncertain,” or “ineffective” and evaluated for accuracy against evidence-based assessments conducted by licensed physicians. We conducted a qualitative content analysis of the response texts and systematically examined the types of sources cited in all responses.

**Results:**

The proportion of Copilot responses claiming supplement effectiveness was 29.4% (53/180), 47.8% (86/180), and 45% (81/180) for the creative, balanced, and precise modes, respectively, whereas overall accuracy of the responses was low across all modes: 36.1% (65/180), 31.7% (57/180), and 31.7% (57/180) for creative, balanced, and precise, respectively. No significant difference was observed among the 3 modes (*P=*.59). Notably, 72.7% (2240/3081) of the citations came from unverified sources such as blogs, sales websites, and social media. Of the 540 responses analyzed, 54 (10%) contained at least 1 citation in which the cited source did not include or support the claim made by Copilot, indicating hallucinated content. Only 48.5% (262/540) of the responses included a recommendation to consult health care professionals. Among disease categories, the highest accuracy was found for cancer-related questions, likely due to lower misinformation prevalence.

**Conclusions:**

This is the first study to assess Copilot’s performance on dietary supplement information. Despite its authoritative appearance, Copilot frequently cited noncredible sources and provided ambiguous or inaccurate information. Its tendency to avoid definitive stances and align with perceived user expectations poses potential risks for health misinformation. These findings highlight the need for integrating health communication principles—such as transparency, audience empowerment, and informed choice—into the development and regulation of AI search engines to ensure safe public use.

## Introduction

### Background

The rise of digital technologies has dramatically transformed how people access and evaluate health information [1]. From a health communication perspective, tools such as artificial intelligence (AI)–powered chatbots play an increasingly important role in shaping how individuals understand and make decisions regarding their health. As these tools become more integrated into everyday search behaviors, examining the accuracy and reliability of the health-related content they provide is crucial [2,3].

One prominent example of such a tool is Microsoft’s AI-powered conversational agent, originally launched as Bing Chat on February 7, 2023 [4]. Later rebranded as Copilot in late 2023, this tool is now integrated across Microsoft platforms, including Bing, Edge, and Windows [5]. Unlike traditional search engines that return a list of hyperlinks, Copilot (formerly Bing Chat) is designed to generate conversational responses by synthesizing information using GPT-4. It can retrieve information from the web in real time, providing users with direct answers that often include reference links [6,7]. Copilot differs from other large language models (LLMs) in several ways. First, unlike other LLMs with a cutoff date [8], it can perform real-time web searches, increasing its risk of incorporating inaccurate or misleading online content [9,10]. Second, it is embedded directly into a widely used search engine, exposing a much larger and more general user base to AI-generated content. On the basis of Bing’s scale, the health communication implications of misinformation are substantial [11]. Third, Copilot provides reference links within its responses that users may perceive as credible and trustworthy, thereby potentially reinforcing inaccuracies [12].

Although Copilot has tens of millions of active users worldwide [13], there is limited research on its reliability in the field of nutrition and dietary planning. One study assessing the accuracy of LLMs in generating kidney-friendly diet plans found that Bing Chat achieved an accuracy of 81%, which is equal to that of GPT-4 and significantly higher than the 66% accuracy of GPT-3.5 [14]. Another study evaluating the ability of LLMs to identify the protein content of foods reported that Bing Chat achieved an accuracy of 63.6%, outperforming GPT-4, which had an accuracy of 60.6% [15]. However, these findings reflect structured and well-established areas of nutritional science. In contrast, the field of dietary supplements is characterized by emerging research, conflicting claims, and a high prevalence of misinformation online [16]. This makes it particularly challenging for real-time web-connected LLMs to generate reliable evidence-based content.

In Japan, approximately 50% of adults report regular or occasional dietary supplement use [17]. Worldwide, the supplement market continues to grow rapidly, with consumers increasingly relying on these products for health maintenance and disease prevention [18]. However, in many countries, dietary supplements are not as strictly regulated as pharmaceuticals, leaving users heavily dependent on internet-based information. A previous study found that the prevalence of misinformation regarding dietary supplements on the internet was significantly higher than that regarding many other health-related domains [18]. Inaccurate information can lead to misinformed health decisions, unnecessary financial costs, and adverse outcomes. Moreover, although Copilot includes reference links in its responses, few studies have examined the trustworthiness of these sources. If misinformation is embedded in an AI-generated summary and in the referenced content, the risk to users is amplified [19].

Therefore, a comprehensive evaluation of Copilot’s reliability in the context of dietary supplement information is crucial for advancing health communication research and supporting safe and informed decision-making in everyday health practices.

### Study Aims and Objectives

We simulated Japanese consumers’ use of Copilot to inquire about dietary supplements to clarify the following issues:

1.  What proportion of Copilot responses characterize a dietary supplement as effective, ineffective, or uncertain?
2.  How does Copilot perform when responding to questions related to various disease categories?
3.  To what extent are Copilot’s responses accurate in the context of dietary supplement information?
4.  What types of sources does Copilot cite in its responses, and how trustworthy are these references?
5.  What types of common errors appear in Copilot’s answers, and how might these inaccuracies mislead users?
6.  Are there any notable differences among responses generated by the 3 different versions of Copilot?

By exploring these research questions, we aimed to understand how internet-based LLMs respond to inquiries about dietary supplements and identify current limitations in their performance. The findings of this study will contribute to the development of more reliable AI in the future and support general consumers in making informed and responsible decisions regarding the use of AI tools.

## Methods

### Dietary Supplement Keywords and Questions

We selected the top 30 dietary supplements by market share in Japan in 2023 as identified in chapter 4, “Present Situation and Prospects of the Health Food Category Market,” of the 2023 edition of Healthy Foods Market Stats and Prospects, Market Survey Edition, published by Yano Research Institute Ltd (Textbox 1) [20]. All the supplements are available in Japan.

Textbox 1. **Keywords of the 30 dietary supplements.**

- *Aojiru*
- *Agaricus*
- *Ginkgo biloba extract*
- *Turmeric*
- *Royal jelly*
- *Ornithine*
- *Oyster extract*
- *Chlorella*
- *Glucosamine*
- *Chitin and chitosan*
- *Ubiquinone*
- *Chinese softshell turtle*
- *Black vinegar*
- *Squalene*
- *Collagen*
- *Oriental ginseng*
- *Soy isoflavone*
- *DHA* and *EHA*
- *Garlic*
- *Lactic acid bacteria*
- *Hyaluronic acid*
- *Vitamin E*
- *Vitamin C*
- *Placenta*
- *Blueberry* and *bilberry*
- *Prune*
- *Propolis*
- *Maca*
- *Euglena*
- *Calcium*

The National Institute of Health and Nutrition in Japan has released evaluations on the effectiveness of numerous dietary supplements across a wide range of health domains (Multimedia Appendix 1) [21]. These systems include the circulatory and respiratory systems, digestive and hepatic systems, endocrine and diabetic conditions, reproductive and urinary systems, brain and sensory functions, immune responses, cancer and inflammatory conditions, musculoskeletal health, developmental processes, and obesity. On the basis of these classifications, we identified 6 common disease areas and developed the corresponding question sets.

For each dietary supplement, 6 questions were generated with reference to a report issued by the National Institute of Health and Nutrition (Textbox 2).

Textbox 2. **The 6 questions presented to Copilot.**

- Question 1: “Is [supplement name, eg, aojiru] effective against cancer?”
- Question 2: “Is [supplement name, eg, aojiru] effective against diabetes?”
- Question 3: “Is [supplement name, eg, aojiru] effective against obesity?”
- Question 4: “Is [supplement name, eg, aojiru] effective against constipation?”
- Question 5: “Is [supplement name, eg, aojiru] effective against joint pain?”
- Question 6: “Is [supplement name, eg, aojiru] effective against hypertension?”

### Tested LLMs and Data Collection

This study evaluated Copilot’s 3 response modes (creative, balanced, and precise), which have since been integrated into a single mode, known as Copilot [22]. The creative, balanced, and precise modes differ primarily in response style. The creative mode tends to generate longer, more exploratory answers; the precise mode produces concise and factual outputs; and the balanced mode lies between the other 2. All modes share the same underlying model and search results [23,24]. The data were collected between July and September 2023. Each question was posed once to each of the 3 Copilot modes, and the generated responses were recorded in a Microsoft Excel spreadsheet. To prevent potential context carryover, the chat window was closed after each query, and a new session was opened before submitting the next question. To ensure that the simulation reflected real-world user scenarios, we did not use prompts in our questions. To assess the reliability of the information sources cited by Copilot, 2 authors (ML and YX) collected all the referenced links from the responses and categorized their source types. Disagreements were resolved through discussion to reach a final consensus.

In addition, to prevent cross-interference between responses related to different dietary supplements, we closed the existing chat after completing the questions for one supplement and initiated a new conversation before proceeding to the next.

### Quantitative Analysis

The reports published by the National Institute of Health and Nutrition in Japan did not directly state whether a given dietary supplement was effective against a specific disease. Instead, a broad range of experimental studies were compiled that examined the effects of each supplement on various diseases. These studies used diverse methodologies, including randomized controlled trials, meta-analyses, and animal experiments. Consequently, the findings for the same supplement-disease pair may vary, with some studies reporting positive effects and others reporting no effects.

To address this variability, 2 licensed Japanese physicians (YN and RS) developed a comprehensive evaluation framework (Multimedia Appendix 2). Using this framework and reports from the National Institute of Health and Nutrition, they assessed the evidence-based effectiveness of the 30 dietary supplements for the 6 diseases. Each outcome was categorized as “effective,” “uncertain,” or “ineffective.” In cases of conflicting evidence, consensus was reached through discussion.

The same two authors (YN and RS) then evaluated the responses generated by Copilot regarding the effectiveness of dietary supplements for the 6 diseases using a separate evaluation guideline they developed (Multimedia Appendix 2). These outcomes were similarly classified as “effective,” “uncertain,” and “ineffective.” The evaluation was conducted in a double-blind manner, and interrater agreement was measured using the Fleiss κ. Disagreements were resolved through consensus.

Finally, the effectiveness stated by Copilot was compared with evidence-based assessments from scientific literature. Responses consistent with the reference assessment were classified as correct; all others were classified as incorrect. All the responses and classification results were recorded in Microsoft Excel (Office 2019 Professional Plus; 64 bits). To determine the statistical significance between groups, 2-tailed *z* tests were conducted [25].

### Qualitative Analysis

In addition to the quantitative assessment of Copilot’s response accuracy, this study used a qualitative approach to analyze the content of the responses. All 540 responses were thoroughly reviewed, and the key characteristics and issues were systematically documented in Microsoft Excel. Common patterns and errors were identified, and relevant excerpts were cited in Japanese with English translations to support our findings.

### Ethical Considerations

All information used in this study was obtained from publicly available sources. Therefore, no ethical approval was required.

## Results

### Evidence-Based Effectiveness of Dietary Supplements

The results of the evaluation of the effectiveness of the 30 dietary supplements for the 6 diseases can be found in Multimedia Appendix 3. The “A,” “B,” and “C” designations correspond to “effective,” “uncertain,” and “ineffective,” respectively. Most dietary supplements had no or uncertain effects on the diseases. Among all supplements, only turmeric and hyaluronic acid were deemed effective for joint pain, whereas black vinegar was deemed effective for hypertension.

### Proposed Effectiveness of Dietary Supplements in Copilot Responses

The Fleiss κ value measuring interrater agreement between the two evaluators was 0.70, indicating substantial consistency. The distribution of responses from the Copilot creative, balanced, and precise modes regarding the effectiveness of the 30 dietary supplements across the 6 diseases is shown in Table 1. Specifically, the proportion of responses indicating that the supplements were “effective” was 29.4% (53/180) for the creative mode, 47.8% (86/180) for the balanced mode, and 45% (81/180) for the precise mode. A statistically significant difference was observed between creative and the other two modes (creative vs balanced: *P*<.001; creative vs precise: *P*=.002; balanced vs precise: *P*=.59). The creative, balanced, and precise modes generated 47.2% (85/180), 30% (54/180), and 30.6% (55/180) of responses categorized as “uncertain,” respectively. Similarly, the difference between creative and the other 2 modes was statistically significant (creative vs balanced: *P*<.001; creative vs precise: *P*=.001; balanced vs precise: *P*=.90). The proportion of responses categorized as “ineffective” was 23.3% (42/180) for the creative mode, 22.2% (40/180) for the balanced mode, and 24.4% (44/180) for the precise mode. No significant differences were observed among the 3 modes in this category (*P*=.88).

**Table 1.** Prevalence of responses from Copilot categorized as “effective,” “uncertain,” and “ineffective” for the 6 diseases.

Response category	Copilot creative mode, n (%)	Copilot balanced mode, n (%)	Copilot precise mode, n (%)
“Is [supplement name, eg, aojiru] effective against cancer?” (n=30)			
Effective	2 (6.7)	7 (23.3)	6 (20)
Uncertain	20 (66.7)	16 (53.3)	15 (50)
Ineffective	8 (26.7)	7 (23.3)	9 (30)
“Is [supplement name] effective against diabetes?” (n=30)			
Effective	4 (13.3)	15 (50)	12 (40)
Uncertain	19 (63.3)	10 (33.3)	14 (46.7)
Ineffective	7 (23.3)	5 (16.7)	4 (13.3)
“Is [supplement name] effective against obesity?” (n=30)			
Effective	9 (30)	17 (56.7)	16 (53.3)
Uncertain	15 (50)	5 (16.7)	6 (20)
Ineffective	6 (20)	8 (26.7)	8 (26.7)
“Is [supplement name] effective against constipation?” (n=30)			
Effective	10 (33.3)	21 (70)	17 (56.7)
Uncertain	15 (50)	6 (20)	4 (13.3)
Ineffective	5 (16.7)	3 (10)	9 (30)
“Is [supplement name] effective against joint pain?” (n=30)			
Effective	9 (30)	10 (33.3)	10 (33.3)
Uncertain	8 (26.7)	9 (30)	9 (30)
Ineffective	13 (43.3)	11 (36.7)	11 (36.7)
“Is [supplement name] effective against hypertension?” (n=30)			
Effective	19 (63.3)	16 (53.3)	20 (66.7)
Uncertain	8 (26.7)	8 (26.7)	7 (23.3)
Ineffective	3 (10)	6 (20)	3 (10)
Total answers (n=180)			
Effective	53 (29.4)	86 (47.8)	81 (45)
Uncertain	85 (47.2)	54 (30)	55 (30.6)
Ineffective	42 (23.3)	40 (22.2)	44 (24.4)

When analyzed by disease, Copilot gave the fewest responses that indicated supplement effectiveness for cancer treatment, with 17/% (15/90) of the responses across all 3 modes. In contrast, the highest proportion of responses suggesting effectiveness was observed for hypertension, with 61% (55/90) of the responses.

Responses indicating supplement ineffectiveness were lowest for hypertension (12/90, 13%) and highest for joint pain (35/90, 39%).

Regarding responses classified as “uncertain,” hypertension had the fewest (23/90, 26%), whereas cancer had the most (51/90, 57% across all modes).

### Accuracy of Copilot Responses

The accuracy rates of the Copilot responses are summarized in Table 2. Overall, the accuracies of the creative, balanced, and precise modes were 36.1% (65/180), 31.7% (57/180), and 31.7% (57/180), respectively, with no significant differences among them (*P*=.59).

When examined by disease category, Copilot showed the highest average accuracy for cancer-related questions (44/90, 49%). In contrast, the lowest accuracy was observed for constipation-related questions with 19% (17/90).

**Table 2.** Correctness of the responses for different diseases.

	Copilot creative mode, n (%)	Copilot balanced mode, n (%)	Copilot precise mode, n (%)
“Is [supplement name] effective against cancer?” (n=30)	17 (56.7)	14 (46.7)	13 (43.3)
“Is [supplement name] effective against diabetes?” (n=30)	13 (43.3)	8 (26.7)	10 (33.3)
“Is [supplement name] effective against obesity?” (n=30)	8 (26.7)	8 (26.7)	7 (23.3)
“Is [supplement name] effective against constipation?” (n=30)	5 (16.7)	3 (10)	9 (30)
“Is [supplement name] effective against joint pain?” (n=30)	16 (53.3)	14 (46.7)	13 (43.3)
“Is [supplement name] effective against hypertension?” (n=30)	6 (20)	11 (36.7)	5 (16.7)
Total (n=180)	65 (36.1)	57 (31.7)	57 (31.7)

### Sources Cited in Copilot Responses

Across 540 responses (3 modes × 30 dietary supplements × 6 diseases), Copilot cited 3081 links, averaging 5.7 sources per response. These sources were categorized into 2 major groups and 14 subcategories (Table 3 and Multimedia Appendix 4). One of the major groups was unverified sources, accounting for 72.7% (2240/3081) of all citations, and included the following subcategories: Bing search page, introduction of food or pharmaceutical sales websites (mainly third-party commercial websites and excluding manufacturers), unregulated medical knowledge websites (eg, blogs or personal articles), Amazon product page for the supplement, other social media platforms (X [formerly known as Twitter], Facebook, YouTube, and Zhihu), and invalid links. The other major group was verified sources, which made up the remaining 27.3% (841/3081) of the citations and included food and pharmaceutical manufacturer websites, hospital and clinic websites, news, Wikipedia, government websites (eg, the Ministry of Health, Labour, and Welfare and local governments), individual research introduction websites (eg, Nature, PubMed, J-GLOBAL, RIKEN, university research highlights, and laboratory websites), pharmacist or medical association websites, and academic conferences.

Among all the subcategories, unregulated medical knowledge websites were the most frequently cited, with 61.4% (1893/3081) of the citations. Other categories with >5% of the total citations included food and pharmaceutical manufacturer websites (241/3081, 7.8% of the citations), Bing search pages (236/3081, 7.7% of the citations), and individual research introduction websites (224/3081, 7.3% of the citations). News, Wikipedia, product sales websites, hospital and clinic websites, and government websites each accounted for 1% to 5% of the citations. Sources such as pharmacist or medical association websites, Amazon, other social media platforms, academic conferences, and invalid links each accounted for <1% of the total citations.

From a disease-specific perspective, the proportion of citations from unregulated medical knowledge websites was highest for obesity and constipation, accounting for 68.9% (367/533) and 69.7% (347/498), respectively. These rates were significantly higher than those for other diseases (cancer vs obesity: *P*<.001; diabetes vs obesity: *P*<.001; joint pain vs obesity: *P*=.007; hypertension vs obesity: *P*=.008; cancer vs constipation: *P*<.001; diabetes vs constipation: *P*<.001; joint pain vs constipation: *P*=.004; hypertension vs constipation: *P*=.004). No significant differences were observed in the citation proportions of other source categories across different diseases.

**Table 3.** Categories of websites cited by Copilot.

Categories	Total, n (%)
Unverified websites	
Bing search page	236 (7.7)
Invalid links	24 (0.8)
Unregulated medical knowledge websites (eg, blogs, personal articles)	1893 (61.4)
Introduction of food/pharmaceutical sales websites (excluding manufacturers)	67 (2.2)
Amazon	11 (0.4)
Other social media platforms (Twitter, Facebook, YouTube, Zhihu)	10 (0.3)
Verified websites	
Food and pharmaceutical manufacture websites	241 (7.8)
Hospital and clinic websites	123 (4.0)
Government websites (eg, Ministry of Health, Labour and Welfare, local governments)	46 (1.5)
Individual research introduction websites (eg, Nature, PubMed, J-Global, RIKEN, university research highlights, lab websites)	224 (7.3)
Pharmacists’ or medical associations websites	13 (0.4)
Academic conferences	7 (0.2)
News	99 (3.2)
Wikipedia	87 (2.8)

### Content Analysis

Copilot responses generally followed a 3-part template. The first section provided a direct answer to the question of whether a specific supplement was effective against a given disease. When expressing a positive stance, Copilot often used highly assertive language, such as “Yes, [supplement] is effective for [disease].” However, when presenting a negative view, it frequently used more ambiguous phrasing—for example, “Although there is no scientific evidence supporting the supplement’s effectiveness for the disease, it may still have potential benefits” or “While it does not act directly on the condition, it might exert indirect effects.”

The second section typically offered a detailed description of the nutritional components of the supplements and their potential physiological effects. This section included citations from several online sources. However, upon reviewing these sources individually, we found that some did not support the claims made in the corresponding Copilot responses.

The third section served as a summary of the overall responses. In many cases, this section partially replicated the opinions stated in the first section. However, this approach often introduced additional statements that diluted or contradicted an initial stance. For example, even if the first section endorsed the supplement’s effectiveness, the summary might include phrases such as “The effects of the supplement may vary from person to person,” “There is no definitive conclusion—some studies support its benefits, while others do not,” or “Excessive intake of the supplement may have adverse effects,” thereby leaning toward a more skeptical or cautious tone. In addition, of the 540 responses, only 262 (48.5%) included a recommendation in the third section for users to consult a health care professional.

## Discussion

### Principal Findings

Our study is the first to comprehensively evaluate an AI search engine’s response quality on supplement-related queries. Overall, the AI search engine cited numerous unverified websites and achieved an accuracy of approximately 33.1% (179/540).

The number of responses indicating that dietary supplements were ineffective was nearly identical across the 3 modes of Copilot: 23.3% (42/180) for the creative mode, 22.2% (40/180) for the balanced mode, and 24.4% (44/180) for the precise mode. The balanced and precise modes yielded a higher number of responses that indicated supplement effectiveness—47.8% (86/180) and 45% (81/180), respectively—whereas the proportion of “uncertain” responses was 30% (54/180) and 30.6% (55/180), respectively. In contrast, the creative mode showed fewer “effective” responses (53/180, 29.4%) and more “uncertain” ones (85/180, 47.2%). Overall, the balanced and precise modes exhibited similar response patterns, whereas the creative mode was more cautious, with a higher proportion of uncertain responses and fewer confident claims of effectiveness.

When comparing Copilot’s reported effectiveness with evidence-based evaluations, both the balanced and precise modes demonstrated an accuracy of 31.7% (57/180), whereas the creative mode achieved a slightly higher accuracy of 36.1% (65/180); however, this difference was not statistically significant (*P*=.38). Generally, the accuracy of all 3 modes was suboptimal, with none exceeding 40%, and all fell well below the accuracy levels reported in previous studies that tested Copilot in the domain of dietary planning and nutrition [14,15].

In addition, we conducted a detailed review and classification of all the cited sources in the Copilot responses, which makes this the first study to systematically analyze citation quality. We found that 72.7% (2240/3081) of the citations came from unverified or nonauthoritative sources, including the Bing search page; introduction of food or pharmaceutical sales websites (excluding manufacturers); unregulated medical knowledge websites (eg, blogs or opinion pieces); Amazon; other social media platforms (X [formerly known as Twitter], Facebook, YouTube, and Zhihu); and invalid links. In contrast, only 27.3% (840/3081) of the citations came from verified and credible sources, such as news; Wikipedia; food and pharmaceutical company websites; hospital and clinic websites; government websites (eg, the Ministry of Health, Labour, and Welfare and local governments); individual research introduction websites (eg, Nature, PubMed, J-GLOBAL, RIKEN, university research highlights, and laboratory websites); pharmacist or medical association websites, and academic conferences. Among all the citation categories, unregulated medical knowledge websites were the most frequently cited across all 3 modes. This finding suggests that the commercial purpose of many unregulated sites makes them more biased and less authoritative as nutritional supplements are over-the-counter consumer items. Moreover, web-scraped datasets often contain advertising content from social media and online articles. Notably, for cancer-related queries, the proportion of such unregulated sources was lower at approximately 54.5% (307/563). In contrast, approximately 79.25% (714/1031) of the sources for obesity (68.9%, 367/533) and constipation (70%, 347/498) fell into this unverified category. This substantial imbalance highlights a key concern in health communication: AI-generated content may present unverified sources in a polished, authoritative format, creating a “credibility illusion” that enhances user trust while disseminating misinformation. This illusion poses a particular risk in public health contexts where information reliability is essential for informed decision-making.

We believe that the low accuracy of Copilot in the field of dietary supplements can be attributed to several key factors. First, dietary planning and nutrition are domains characterized by well-structured knowledge with clearly established guidelines and recommendations grounded in scientific consensus. Questions in nutrition and medical areas tend to have definitive answers and logical reasoning paths that align well with the strengths of LLMs [26-30]. However, the effectiveness of dietary supplements remains a subject of scientific controversy [31,32]. For example, studies on glucosamine’s effects on joint health include both positive and negative findings even among meta-analyses [31,32]. Second, the prevalence of inaccurate or misleading information is significantly lower in the dietary planning and nutrition domains than in the dietary supplement domain [18,33]. The former is typically documented in textbooks, peer-reviewed literature, and official guidelines, whereas the latter often includes a wide range of unverified claims from advertisements and personal blogs [18,33]. Our study found that 72.7% (2240/3081) of the sources cited by Copilot were from unverified websites. Because the internet serves as a major component of LLM training data, the abundance of unverified content likely affects the model’s response accuracy in this domain. Third, unlike ChatGPT, which screens and synthesizes information from its training data, Bing is fundamentally a search engine that quotes content directly from web pages. Consequently, erroneous or misleading information may be presented to users without screening. Notably, we observed that the creative mode achieved higher accuracy than the balanced and precise modes. This may be due to its higher-temperature setting, which allows for more exploratory reasoning and the integration of information from multiple sources [34]. Paradoxically, when training data contain a large volume of inaccurate or conflicting content, as is common in the dietary supplement domain, a higher temperature may enable the model to reason beyond dominant but incorrect narratives, thus improving the response quality [34]. In contrast, the balanced and precise modes may rely more heavily on conservative surface-level content from citations, leading to less accurate responses. Therefore, we hypothesized that, in complex and controversial domains, higher-temperature models may perform better by generating responses through more bold and exploratory reasoning and a broader synthesis of information.

Across the different disease categories, all 3 Copilot modes provided the fewest responses that indicated the effectiveness of dietary supplements for cancer treatment and achieved the highest accuracy for this disease. One possible explanation is that, owing to the complexity and severity of cancer, medical information related to its treatment is subject to strict scrutiny and regulation [35-37]. Compared with compared with other health topics, for other conditions, there is a growing consensus among authoritative organizations and scientific literature that emphasizes the lack of credible evidence supporting the use of dietary supplements in cancer therapy [35-37]. As Copilot is trained on such reliable sources, it is more likely to adopt a cautious stance when addressing cancer-related questions. Furthermore, a Japanese study found no advertisements promoting dietary supplements as effective cancer treatments, suggesting that cancer—being a serious and life-threatening disease—is rarely the focus of supplement marketing [38]. Consequently, misinformation about supplements in the context of cancer is likely to be less prevalent than in other disease areas. This relative scarcity of misleading information in the training data may reduce the chances of LLMs incorrectly asserting that supplements are effective against cancer.

In addition to quantitative analysis, we conducted a thorough qualitative review of Copilot responses and found that they followed a highly templated structure: the first section provided a 1-sentence answer to the question, the second section elaborated on the components of the dietary supplement and their potential effects, and the third section offered a summary.

In the first section, Copilot consistently leaned toward affirming, or at least not fully denying, the effectiveness of dietary supplements. Even when initially rejecting the efficacy of a supplement, the response was often followed by hedging statements such as “it may still have some benefits” or “it could exert indirect effects.” We interpret this as a result of the model’s tendency to align its responses with the users’ implicit expectations by providing more positive information. This behavior is consistent with the well-documented phenomenon of sycophancy in LLMs, where the model adjusts its outputs to reflect the tone or assumptions of user inputs, sometimes at the expense of factual accuracy [39,40]. This tendency is particularly problematic in domains such as dietary supplements, where scientific evidence is often inconclusive or disputed. Ambiguous responses catering to user expectations may inadvertently mislead consumers. Therefore, we recommend that future model updates prioritize reducing such ambiguity to minimize the risk of misinformation and improve the reliability of health-related AI responses.

In the second section, Copilot typically cited several websites to support the claims made in the first section. However, 2 notable and concerning patterns were identified. First, in most cases, the model directly repeated claims from the cited sources without conducting any meaningful synthesis or critical evaluation. Consequently, misleading or inaccurate information from nonauthoritative websites was often presented to users without a filter. Second, upon reviewing the linked content individually, we discovered that approximately 9.6% (52/540) of the responses included fabricated claims—statements in Copilot’s responses were attributed to sources that did not contain such information. In most of these cases, Copilot suggested a health effect of a dietary supplement, yet the cited link provided no evidence or mention of that specific claim. Copilot likely exhibited AI hallucinations, a phenomenon in which the model generates content that appears plausible but is factually incorrect or entirely fabricated [41-43]. In these cases, the model appears to have first “invented” a claim about a supplement’s effect and then “fabricated” support for that claim by attaching existing but unrelated or irrelevant citations. The citation itself is real; however, the information it contains does not support the model’s opinion [44].

In the third section, we observed that many responses diluted the affirmative claims presented in the first section. For instance, the model frequently added statements such as “the effects may vary between individuals” or “excessive intake may cause adverse effects.” This suggests that Copilot adopts a cautious and self-protective response strategy characterized by compromise and hedging. This conservative generation strategy is particularly prevalent when dealing with sensitive topics such as health, medicine, and nutrition [45]. Additionally, we found that only 48.5% (262/540) of all responses included recommendations for users to consult health care professionals. We believe that, for medicine-related queries, it would be more appropriate to universally include recommendations for users to follow professional medical advice.

From a health communication perspective, our findings underscore the dual role of internet-based AI tools such as Copilot in shaping the public understanding of health information. On the one hand, AI-generated responses can present complex medical content in a simplified and accessible manner, potentially lowering barriers to health literacy and supporting informed decision-making, particularly among populations with limited access to professional health care resources. However, our analysis revealed that Copilot frequently cites unverified sources and uses ambiguous or overly agreeable language when addressing health-related queries. This may contribute to a “credibility illusion,” whereby users perceive AI-generated content as trustworthy owing to its polished presentation and apparent authority regardless of its actual evidentiary basis. In domains such as dietary supplements, where scientific evidence is frequently inconclusive and commercial interest is strong, this illusion poses a significant risk of misinformation. Moreover, such dynamics can exacerbate existing health information asymmetries by disproportionately affecting users who cannot critically assess online content quality. These findings highlight the urgent need to embed the core principles of health communication, such as informed choice, audience empowerment, and transparency, into the design and governance of AI systems. Only through such efforts can these technologies fulfill their promise as facilitators of public health rather than inadvertent amplifiers of health misinformation.

### Limitations

This study evaluated Copilot’s creative, balanced, and precise modes, which were later merged into a single default mode. Reports suggest that the 3 modes mainly differed in style parameters (eg, verbosity and creativity) rather than underlying model architecture. Although explicit mode switches have been removed, the unified mode likely preserves a blended style; therefore, our accuracy and citation results remain informative [23,24].

Second, this study focused on evaluating Copilot’s performance in a Japanese-language context and did not assess its accuracy in other languages or cultural settings. As a multilingual tool, the performance of Copilot may vary depending on linguistic and cultural factors, as well as on the distribution of its training data. Given that English is likely to be overrepresented in the training corpus, responses in non-English languages such as Japanese may be less accurate because of limited high-quality data.

### Conclusions

This study is the first to evaluate the performance of an AI search engine in the dietary supplement domain. Overall, the results were suboptimal. Copilot affirmed the effectiveness of dietary supplements in approximately 40.7% (220/540) of the responses, yet the overall accuracy was only approximately 33.1% (179/540). The creative mode performed slightly better than the others, achieving an accuracy of 36.1% (65/180), suggesting that higher-temperature LLMs may perform better in complex domains. The primary challenge for Copilot seems to arise from the controversial and inconclusive nature of scientific evidence regarding dietary supplements. Notably, 72.7% (2240/3081) of the sources cited, such as personal blogs and sales websites, were unverified. Copilot frequently quoted information from these sources without proper screening and, in some cases, even attributed claims to sources that did not support them. In terms of response style, Copilot tended to adopt a conservative and hedging tone, often avoiding a clear affirmation or denial of supplement effectiveness. This reflects a tendency toward sycophancy, in which responses align with perceived user expectations. Finally, only half (262/540, 48.5%) of the responses included recommendations to consult medical professionals. We believe that all health-related answers should include such guidance to ensure user safety and responsibility.

## Supplementary material

10.2196/78436 Multimedia Appendix 1 An example of effectiveness reports on dietary supplements for various body systems published by the National Institute of Health and Nutrition in Japan.

10.2196/78436 Multimedia Appendix 2 Evaluation framework for evidence-based dietary supplement effects.

10.2196/78436 Multimedia Appendix 3 Evidence-based effects of 30 dietary supplements for 6 diseases.

10.2196/78436 Multimedia Appendix 4 Categories of the sources cited by Copilot.

## Abbreviations

AI: artificial intelligence
LLM: large language model
