# Supplementary material for: Evaluating the Reliability and Accuracy of an AI-Powered Search Engine in Providing Responses on Dietary Supplements: Quantitative and Qualitative Evaluation
Source: JMIR AI. 2025 Oct 29;4:e78436. doi: 10.2196/78436 (PMC12571200; doi:10.2196/78436)
Supplement: Multimedia Appendix 1 [file ai-v4-e78436-s001.docx]

**Supplementary Materials 1. An example of effectiveness reports dietary supplements for various body system published by National Institute of Health and Nutrition in Japan.**

**Translated version in English.**

| **Item** | **Information** |
| --- | --- |
| **Name** | Agaricus, Himematsutake, Mematsutake, Kawariharatake, Agaricus blazei  [English] Agaricus [Scientific Name] Agaricus blazei Murill |
| **Overview** | Agaricus is a type of mushroom that grows from the ground, featuring a long, thick stem and a strong aroma. It is also known as Agaricus blazei or Himematsutake. Since its introduction from Brazil in 1965, it has been cultivated artificially. However, its characteristics and components vary depending on the strain, cultivation conditions, and production region. |
| **Regulations & Legal Classification** | - Food and Drug Classification:   - Fruiting body: It is classified as a "natural ingredient (raw material) that is not considered a pharmaceutical product unless it claims medicinal effects." |
| **Characteristics & Quality of Components** | |
| **Main Components & Properties** | - Compared to other mushrooms, Agaricus contains a higher amount of crude protein. - It has been reported to contain polysaccharides, vitamins B1, B2, niacin, B6, biotin, pantothenic acid, folic acid, amino acids, vitamin D, magnesium, and potassium (PMID: 18604247). - The β-glucan content in Himematsutake extracts and commercial Agaricus products varies among products (2005215401). - The agaritine content in Himematsutake has been reported to range from 500 to 5,000 mg/kg (dry weight) (PMID: 19680875). - Some Agaricus products have been found to contain high levels of cadmium, but voluntary standards have been implemented to address this issue (104). - Due to the significant variation in quality among Agaricus-containing products, certain voluntary guidelines require overdose testing in humans (PMID: 18408335). |
| **Analysis Methods** | - Agaricus, like other mushroom and yeast products, contains β-D-glucan, but the structural characteristics and molecular weight distribution differ significantly depending on the mushroom type. There is no consensus on the relationship between structure and activity. The total β-glucan content in mushrooms has been measured using a specific detection kit (2005215401). - Ergosterol, a vitamin D precursor found in mushrooms, has been analyzed using gas chromatography-mass spectrometry (GC-MS) (PMID: 9872771). - The analysis of agaritine and its derivatives has been conducted using HPLC-fluorescence detection (PMID: 16755074) and LC-electrospray ionization tandem mass spectrometry (PMID: 17071520). |
| **Efficacy & Clinical Evaluation in Humans** | |
| **Cardiovascular & Respiratory System** | - Randomized Controlled Trial (RCT)   - A double-blind, randomized, placebo-controlled trial conducted in Japan on 33 individuals with mild hypertension examined the effects of consuming Agaricus granules (2g, twice a day, n=10, mean age: 49.3±10.7 years) or Agaricus extract (50 mL, twice a day, n=12, mean age: 47.4±9.0 years) for four weeks. No significant changes were observed in systolic/diastolic blood pressure, pulse rate, body weight, BMI, or biochemical markers (2007007822). |
| **Digestive System & Liver** | No relevant studies were found in the reviewed literature. |
| **Diabetes & Endocrine System** | - RCT   - A double-blind, randomized, placebo-controlled trial conducted in China involved 60 participants with type 2 diabetes who had been on Gliclazide and Metformin for over six months. The trial examined the effects of Agaricus extract (1,500 mg/day, content unspecified) for 12 weeks. A significant reduction in the HOMA-IR marker (insulin resistance index) was observed, but no effects on fasting blood glucose, HbA1c, insulin, or adiponectin levels were found (PMID: 17309383). |
| **Reproductive & Urinary System** | No relevant studies were found in the reviewed literature. |
| **Neurological & Sensory System** | No relevant studies were found in the reviewed literature. |
| **Immune System, Cancer & Inflammation** | - RCT   - A double-blind, crossover, placebo-controlled trial in Japan investigated the effects of Agaricus (A. brasiliensis KA21) at 3 g/day for 7 days in eight healthy adults (mean age: 22.3±0.5 years). Results showed a significant increase in NK (natural killer) cell activity against K562 cells, but no change in NK cell count (PMID: 18604247).   - Another double-blind, randomized, placebo-controlled trial in Brazil involved 57 healthy elderly women (test group: 28 participants, mean age: 70.4±6.3 years). Participants consumed Agaricus dried extract (300 mg, three times a day) for 60 days. No significant effects were observed on BMI, waist circumference, blood pressure, or cytokine levels (IL-6, IFN-γ, TNF-α) (PMID: 22010847). |
| **Skeletal & Muscular System** | No relevant studies were found in the reviewed literature. |
| **Growth & Development** | No relevant studies were found in the reviewed literature. |
| **Obesity** | No relevant studies were found in the reviewed literature. |
| **Other Conditions** | No relevant studies were found in the reviewed literature. |
| **References** | |
| **(2005215401) 東京都立衛生研究所研究年報.2002;53:165-8.**  **(PMID:12204626) Carbohydr Res. 2002 Sep 3;337(15):1417-21.**  **(PMID:9872771) Appl Environ Microbiol. 1999 Jan;65(1):138-42.**  **(104) 「担子菌類中の有害物質の評価に関する研究」（平成１６年度厚生労働科学 研究報告書）**  **(PMID:15833380) Food Chem Toxicol. 2005 Jul;43(7):1047-53.**  **(PMID:17105737) Jpn J Clin Oncol. 2006 Dec;36(12):808-10.**  **(2005013083) 老年消化器病. 2004;16（2）:101-7.**  **(2004276900) 皮膚病診療. 2004;26（8）:967-70.**  **(2004252918) Minophagen Med Rev. 2004;49(3):176-8.**  **(2004149979) 肝臓. 2004;45(2):96-108. (2004125341) 日本胸部臨床. 2003;62(11):1027-31. (2005001969) 肺癌. 2004;44(3):167-71.**  **(PMID:17309383) J Altern Complement Med. 2007 Jan-Feb;13(1):97-102.**  **(2006138431) プラクティス. 2006;23(1):89-92.**  **(2006113539) 臨床神経学. 2006;46(1):67.**  **(2006035857) 臨床皮膚科. 2005;59(10):1013-5.**  **(2006209905) 皮膚の科学. 2006;5(1):102.**  **(PMID:16755074) Chem Pharm Bull (Tokyo). 2006 Jun;54(6):922-4.**  **(PMID:17071520) Food Addit Contam. 2006 Nov;23(11):1179-86.**  **(PMID:7737599) Food Chem Toxicol. 1995 Apr;33(4):257-64.**  **(PMID:9276636) Carcinogenesis. 1997 Aug;18(8):1603-8.**  **(PMID:17707568) Food Chem Toxicol. 2008 Jan;46(1):87-95.**  **(PMID:9571772) Biosci Biotechnol Biochem. 1998 Mar;62(3):434-7**  **(PMID:18154438) JARMAM 臨床微生物迅速診断研究会誌.2007;18(2):103-7. (PMID:18353517) Food Chem Toxicol. 2008 Jun;46(6):1949-59.**  **(PMID:19170155) Phytother Res. 2009 Jul;23(7):906-12.**  **(PMID:19879310) Food Chem Toxicol. 2010 Jan;48(1):402-8.**  **(PMID:20649730) J Dermatol. 2010 Aug;37(8):773-5.**  **(PMID:22010847) Scand J Immunol. 2012 Mar;75(3):336-41. (2010342411) 糖尿病. 2010;53(8):647. (2010017845) 西日本泌尿器科. 2009;71:144.**  **(PMID:19680875) Food Addit Contam Part A Chem Anal Control Expo Risk A ssess. 2009 Jan;26(1):82-93.**  **(PMID:9625538) Cancer Immunol Immunother. 1998 May;46(3):147-59.**  **(PMID:12224654) Biosci Biotechnol Biochem. 2002 Jul;66(7):1610-4.**  **(22) メディカルハーブ安全性ハンドブック第2版東京堂出版林真一郎ら監訳**  **(2014145301) Clinical Journal of Gastroenterology. 2013; 6(2): 139-44.**  **(91) Registry of Toxic Effects of Chemical Substances (RTECS) (2007007822) Health Sci. 2006;22(3):316-28.**  **(2012341380) J Environ Dermatol Cutan Allergol. 2012;6(3):277.**  **(2007343698) 日本腎臓学会誌. 2007;49(6):594.**  **(PMID:18604247) Evid Based Complement Alternat Med. 2008 J un;5(2):205-19. (PMID:18408335) 日本薬理学雑誌.2008 Apr;131(4):252-7.**  **(2005052247) 日本癌治療学会誌. 2004:39(2);426.**  **(2018108228) 日本呼吸器学会誌. 2017:6(3);186-9.**  **(30) 「医薬品の範囲に関する基準」(別添1、別添2、一部改正について)** | |

**
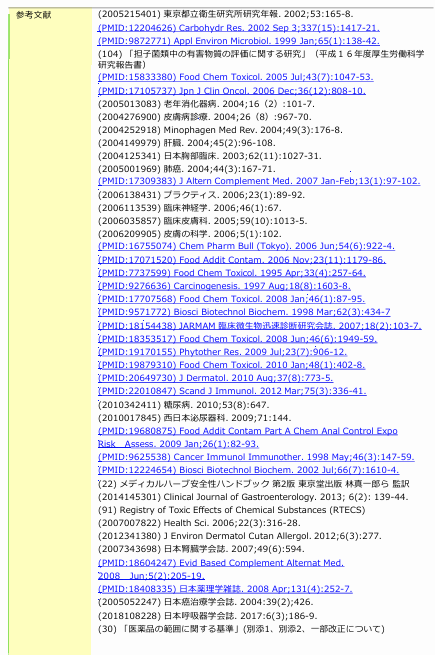

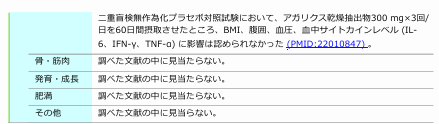

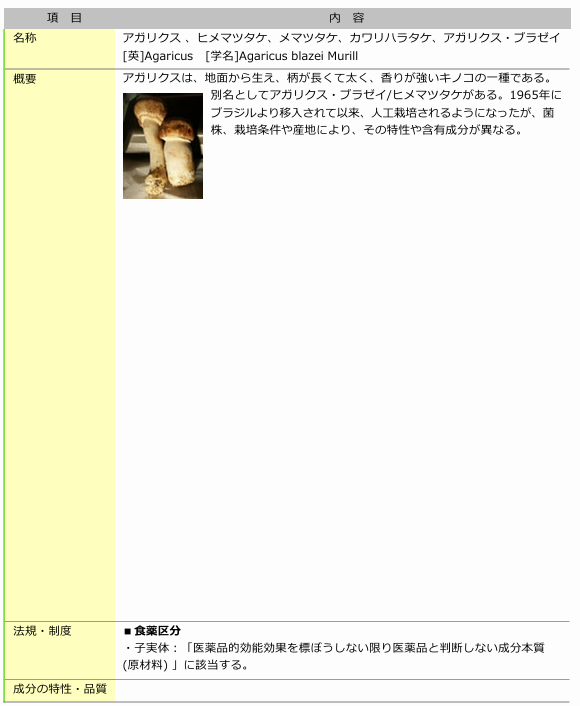
Original version in Japanese.**

**
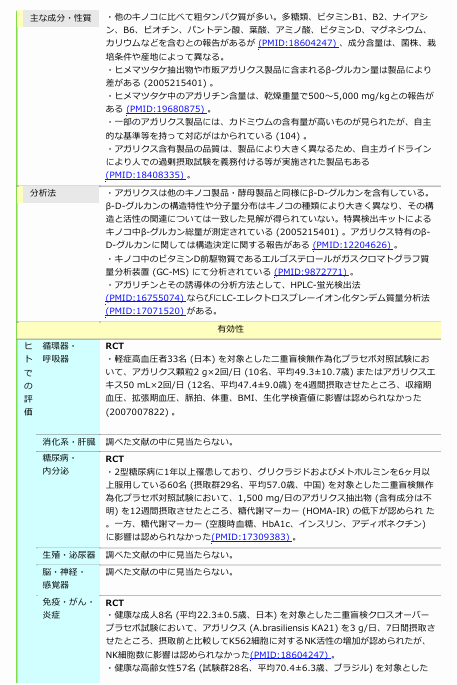
**
