# Supplementary material for: Evaluating the Reliability and Accuracy of an AI-Powered Search Engine in Providing Responses on Dietary Supplements: Quantitative and Qualitative Evaluation
Source: JMIR AI. 2025 Oct 29;4:e78436. doi: 10.2196/78436 (PMC12571200; doi:10.2196/78436)
Supplement: Multimedia Appendix 2 [file ai-v4-e78436-s002.docx]

**Supplementary materials 2. Evaluation framework for evidence-based effects.**

|  | Effective | Uncertain | Not effective |
| --- | --- | --- | --- |
| Cancer | For cancer patients: 1. Extension of overall survival time 2. Disease-free survival time 3. Symptom improvement 4. Objective response rate 5. Progression-free survival time For healthy adults: 1. Reduction in incidence/mortality risk as shown in meta-analyses, RCTs, or prospective cohort studies. | 1. Effects at the vitro test level (e.g., T-cell, NK-cell studies). 2. Effects observed in animal experiments. 3. Reduction in incidence/mortality risk observed in studies other than meta-analyses, RCTs, or prospective cohort studies (e.g., case-control studies). 4. Theoretical references to potential effects (e.g., reduction of oxidative stress, anti-inflammatory effects). 5. Simultaneous reports of positive and negative results. | 1. No research exists, and the effect is considered unlikely, even theoretically. 2. Comparative trials on direct effect indicators concluded no effect. |
| Diabetes | 1. Improvement in fasting blood glucose levels. 2. Improvement in HbA1c. 3. Improvement in insulin resistance markers (e.g., HOMR-IR × fasting insulin levels). 4. Improvement in insulin secretion indicators (e.g., 75g OGTT, blood insulin/C-peptide levels post-meal, daily C-peptide levels in urine). 5. Pancreatic β-cell function evaluation indicators (e.g., HOMA-β, proinsulin/insulin ratio, proinsulin/C-peptide ratio). 6. Physical measurements (e.g., BMI, blood pressure, waist circumference). 7. Improvement in complications (e.g., nephropathy, retinopathy, neuropathy, atherosclerosis indicators). 8. Improved perinatal outcomes in gestational diabetes (e.g., reduced fetal death, large babies, cesarean sections, shoulder dystocia, neonatal respiratory distress syndrome) and decreased risk of postpartum diabetes onset. 9. Reduction in incidence/mortality risk based on meta-analyses, RCTs, or prospective cohort studies (for healthy adults). | 1. Effects observed in vitro testing. 2. Effects observed in animal studies. 3. Theoretical references to potential effects (e.g., anti-inflammatory effects improving insulin resistance). 4. Simultaneous reports of positive and negative results. 5. Reduction in incidence/mortality risk observed in studies other than meta-analyses, RCTs, or prospective cohort studies (e.g., case-control studies, for healthy adults). | 1. No research exists, and the effect is considered unlikely, even theoretically. 2. Comparative trials on direct effect indicators concluded no effect. |
| Obesity | 1. Weight reduction and BMI improvement. 2. Decrease in visceral fat and waist circumference. 3. Improvement in obesity-related health disorders (e.g., diabetes, hypertension). | 1. Effects observed in vitro testing. 2. Effects observed in animal studies. 3. Theoretical references to potential effects. 4. Simultaneous reports of positive and negative results. | 1. No research exists, and the effect is considered unlikely, even theoretically. 2. Comparative trials on direct effect indicators concluded no effect. |
| Constipation | 1. Reduction in straining during defecation. 2. Improvement in stool consistency (BSFS). 3. Improvement in residual stool sensation. 4. Improvement in defecation difficulty. 5. Reduction in manual defecation assistance. 6. Increase in spontaneous bowel movements. | 1. Effects observed in vitro testing. 2. Effects observed in animal studies. 3. Theoretical references to potential effects (e.g., increased bile secretion, regulation of intestinal immunity). 4. Simultaneous reports of positive and negative results. | 1. No research exists, and the effect is considered unlikely, even theoretically. 2. Comparative trials on direct effect indicators concluded no effect. |
| Joint pain | 1. Pain improvement. | 1. Effects observed in vitro testing. 2. Effects observed in animal studies. 3. Theoretical references to potential effects. 4. Simultaneous reports of positive and negative results. | 1. No research exists, and the effect is considered unlikely, even theoretically. 2. Comparative trials on direct effect indicators concluded no effect. |
| Hypertension | 1. Reduction in systolic blood pressure. 2. Reduction in diastolic blood pressure. | 1. Effects observed in vitro testing. 2. Effects observed in animal studies. 3. Theoretical references to potential effects. 4. Simultaneous reports of positive and negative results. | 1. No research exists, and the effect is considered unlikely, even theoretically. 2. Comparative trials on direct effect indicators concluded no effect. |

**The guideline used to evaluate the effectiveness of dietary supplements for specific diseases as presented in the Copilot responses**

| **Category** | **Definition** | **Examples** |
| --- | --- | --- |
| **A** | The text consists of statements affirming the direct or indirect effects of the supplement. No mention of denying effectiveness.  - | - Some studies show effectiveness.  - There are indirect effects.  - There are still few studies demonstrating effectiveness, but it is promising. |
| **B** | The text consists of ambiguous statements, such as both positive and negative statements about the effect, or insufficient scientific evidence for the effect, although the effect is positive. | - Mentions both effectiveness and lack of effectiveness.  - Acknowledges indirect effects but questions direct effects.  - There is some scientific evidence of effectiveness, but it is insufficient or not fully established.  - There are individual differences.  - The independent effect is limited, but when combined with treatments, it may be effective (supplementary effect). |
| **C** | The text consists of statements that question or do not mention the effect on the disease. | - It is not effective, but it is important for health.  - Effectiveness is questioned.  - Scientific evidence of effectiveness is limited.  - It does not have an effect on constipation, but it may improve gut health.  - There is little evidence that it relieves joint pain, but it helps maintain joint health. |

| **Supplement name** | **URL of each dietary supplement of National Institute of Health and Nutrition** |
| --- | --- |
| Aojiru | https://hfnet.nibn.go.jp/material-infodb/material-data/%e9%9d%92%e6%b1%81/?tab=general |
| Turmeric | https://hfnet.nibn.go.jp/material-infodb/validity-infodb/ |
| Oyster Extract | https://hfnet.nibn.go.jp/material-infodb/material-data/%e3%82%ab%e3%82%ad%e6%8a%bd%e5%87%ba%e7%89%a9%ef%bc%88%e7%89%a1%e8%a0%a3%e6%8a%bd%e5%87%ba%e7%89%a9%ef%bc%89/?tab=general |
| Chitin & Chitosan | https://hfnet.nibn.go.jp/material-infodb/material-data/%e3%82%ad%e3%83%88%e3%82%b5%e3%83%b3/?tab=general |
| Black Vinegar | https://hfnet.nibn.go.jp/material-infodb/material-data/%e9%bb%92%e9%85%a2/?tab=general |
| Oriental ginseng | https://hfnet.nibn.go.jp/material-infodb/material-data/%e6%9c%9d%e9%ae%ae%e3%83%8b%e3%83%b3%e3%82%b8%e3%83%b3%e3%80%81%e3%82%aa%e3%82%bf%e3%83%8d%e3%83%8b%e3%83%b3%e3%82%b8%e3%83%b3%e3%80%81%e9%ab%98%e9%ba%97%e4%ba%ba%e5%8f%82/?tab=general |
| Garlic | https://hfnet.nibn.go.jp/material-infodb/material-data/%e3%83%8b%e3%83%b3%e3%83%8b%e3%82%af%e3%80%81%e3%82%bb%e3%82%a4%e3%83%a8%e3%82%a6%e3%83%8b%e3%83%b3%e3%83%8b%e3%82%af%e3%80%81%e3%82%ac%e3%83%bc%e3%83%aa%e3%83%83%e3%82%af%e3%80%81%e3%82%aa%e3%82%aa/?tab=general |
| Vitamin E | https://www.nibiohn.go.jp/eiken/info/pdf/k068.pdf |
| Agaricus | <https://hfnet.nibn.go.jp/material-infodb/material-data/%e3%82%a2%e3%82%ac%e3%83%aa%e3%82%af%e3%82%b9%e3%80%81%e3%83%92%e3%83%a1%e3%83%9e%e3%83%84%e3%82%bf%e3%82%b1%e3%80%81%e3%83%a1%e3%83%9e%e3%83%84%e3%82%bf%e3%82%b1%e3%80%81%e3%82%ab%e3%83%af%e3%83%aa/?tab=general> |
| Royal jelly | https://hfnet.nibn.go.jp/material-infodb/material-data/%e3%83%ad%e3%83%bc%e3%83%a4%e3%83%ab%e3%82%bc%e3%83%aa%e3%83%bc/?tab=general |
| Chlorella | https://hfnet.nibn.go.jp/material-infodb/material-data/%e3%82%af%e3%83%ad%e3%83%ac%e3%83%a9/?tab=general |
| Ubiquinone (UQ) | https://hfnet.nibn.go.jp/material-infodb/material-data/%e3%82%b3%e3%82%a8%e3%83%b3%e3%82%b6%e3%82%a4%e3%83%a0q10%e3%80%81%e3%83%a6%e3%83%93%e3%82%ad%e3%83%8e%e3%83%b3/?tab=general |
| Squalene | https://www.nibiohn.go.jp/eiken/info/pdf/k223.pdf |
| Soy Isoflavone | https://hfnet.nibn.go.jp/material-infodb/material-data/%e3%83%80%e3%82%a4%e3%82%ba%e3%82%a4%e3%82%bd%e3%83%95%e3%83%a9%e3%83%9c%e3%83%b3/?tab=general |
| Lactic acid bacteria | https://hfnet.nibn.go.jp/material-infodb/material-data/%e4%b9%b3%e9%85%b8%e8%8f%8c/?tab=general |
| Vitamin C | https://www.nibiohn.go.jp/eiken/info/pdf/k018.pdf |
| Ginkgo biloba extract | https://hfnet.nibn.go.jp/material-infodb/material-data/%e3%82%a4%e3%83%81%e3%83%a7%e3%82%a6%e8%91%89%e3%82%a8%e3%82%ad%e3%82%b9/?tab=general |
| Ornithine | https://hfnet.nibn.go.jp/material-infodb/material-data/%e3%82%aa%e3%83%ab%e3%83%8b%e3%83%81%e3%83%b3/?tab=general |
| Glucosamine | https://hfnet.nibn.go.jp/material-infodb/material-data/%e3%82%b0%e3%83%ab%e3%82%b3%e3%82%b5%e3%83%9f%e3%83%b3/?tab=general |
| Chinese softshell turtle | https://www.nibn.go.jp/eiken/info/pdf/k339.pdf |
| Collagen | https://hfnet.nibn.go.jp/material-infodb/material-data/%e3%82%b3%e3%83%a9%e3%83%bc%e3%82%b2%e3%83%b3/?tab=general |
| DHA & EHA | https://www.nibiohn.go.jp/eiken/info/pdf/k045.pdf |
| Hyaluronic acid | https://hfnet.nibn.go.jp/material-infodb/material-data/%e3%83%92%e3%82%a2%e3%83%ab%e3%83%ad%e3%83%b3%e9%85%b8/?tab=general |
| Placenta | https://hfnet.nibn.go.jp/material-infodb/material-data/%e3%83%97%e3%83%a9%e3%82%bb%e3%83%b3%e3%82%bf%e3%80%81%e8%83%8e%e7%9b%a4/?tab=general |
| Blueberry & Bilberry | https://hfnet.nibn.go.jp/material-infodb/material-data/%e3%83%96%e3%83%ab%e3%83%bc%e3%83%99%e3%83%aa%e3%83%bc/?tab=general |
| Maca | https://hfnet.nibn.go.jp/material-infodb/material-data/%e3%83%9e%e3%82%ab/?tab=general |
| Prune | https://hfnet.nibn.go.jp/material-infodb/material-data/%e3%82%bb%e3%82%a4%e3%83%a8%e3%82%a6%e3%82%b9%e3%83%a2%e3%83%a2%e3%80%81%e3%83%97%e3%83%ab%e3%83%bc%e3%83%b3/?tab=general |
| Euglena | https://hfnet.nibn.go.jp/material-infodb/material-data/%e3%83%9f%e3%83%89%e3%83%aa%e3%83%a0%e3%82%b7%e3%80%81%e3%83%a6%e3%83%bc%e3%82%b0%e3%83%ac%e3%83%8a/?tab=general |
| Propolis | https://hfnet.nibn.go.jp/material-infodb/material-data/%e3%83%97%e3%83%ad%e3%83%9d%e3%83%aa%e3%82%b9/?tab=general |
| Calcium | https://www.nibiohn.go.jp/eiken/info/pdf/k030.pdf |
