# Supplementary material for: Evaluating the Reliability and Accuracy of an AI-Powered Search Engine in Providing Responses on Dietary Supplements: Quantitative and Qualitative Evaluation
Source: JMIR AI. 2025 Oct 29;4:e78436. doi: 10.2196/78436 (PMC12571200; doi:10.2196/78436)
Supplement: Multimedia Appendix 3 [file ai-v4-e78436-s003.docx]

**Supplementary materials 3. Evidence based effects of 30 dietary supplements for six diseases.**

| Dietary supplements name | Cancer | Diabetes | Obesity | Constipation | Joint pain | Hypertension |
| --- | --- | --- | --- | --- | --- | --- |
| Aojiru | C | C | C | C | C | C |
| Agaricus | B | B | C | C | C | C |
| Ginkgo Biloba | C | C | C | C | C | B |
| Turmeric | C | C | C | C | A | C |
| Ornithine | C | C | B | C | C | C |
| Oyster Extract | C | C | C | C | C | C |
| Chitin and Chitosan | C | C | B | C | C | B |
| Glucosamine | B | C | C | C | B | C |
| Black Vinegar | C | C | C | C | C | A |
| Chlorella | B | C | C | C | C | C |
| Korean Ginseng | B | B | C | C | C | C |
| Coenzyme Q10 | B | C | C | C | C | B |
| Collagen | C | C | C | C | B | C |
| Squalene | C | C | C | C | C | C |
| Chinese softshell turtle | C | C | C | C | C | C |
| Soy Isoflavone | B | B | B | C | C | B |
| DHA & EHA | B | B | B | C | C | B |
| Lactic acid bacteria | C | C | B | C | C | C |
| Garlic | B | B | C | C | B | B |
| Hyaluronic acid | C | C | C | C | A | C |
| Vitamin E | B | C | C | C | C | C |
| Vitamin C | B | C | C | C | C | B |
| Placenta | C | C | C | C | C | C |
| Blueberry & Bilberry | C | B | C | C | C | C |
| Prune | C | C | C | C | C | C |
| Propolis | C | B | C | C | C | C |
| Maca | C | C | C | C | C | C |
| Euglena | C | C | C | C | C | C |
| Royal jelly | C | B | C | C | C | B |
| Calcium | B | B | B | C | C | C |

**Legend: A = Effective, B = Uncertain, C = Ineffective**
